# 6-*n*-Butoxy-10-nitro-12,13-dioxa-11-azatricyclo[7.3.1.0^2,7^]trideca-2,4,6,10-tetraene Improves the X-ray Sensitivity on Inhibiting Proliferation and Promoting Oxidative Stress and Apoptosis of Oral Cancer Cells

**DOI:** 10.3390/biomedicines12020458

**Published:** 2024-02-19

**Authors:** Kun-Han Yang, Ching-Yu Yen, Sheng-Chieh Wang, Fang-Rong Chang, Meng-Yang Chang, Chieh-Kai Chan, Jiiang-Huei Jeng, Jen-Yang Tang, Hsueh-Wei Chang

**Affiliations:** 1 Graduate Institute of Natural Products, Kaohsiung Medical University, Kaohsiung 80708, Taiwan; u110831001@kmu.edu.tw (K.-H.Y.); aaronfrc@kmu.edu.tw (F.-R.C.); 2 School of Dentistry, Taipei Medical University, Taipei 11031, Taiwan; ycy@tmu.edu.tw; 3 Department of Oral and Maxillofacial Surgery, Chi-Mei Medical Center, Tainan 71004, Taiwan; 4 Department of Biomedical Science and Environmental Biology, PhD Program in Life Sciences, College of Life Science, Kaohsiung Medical University, Kaohsiung 80708, Taiwan; u107851101@gap.kmu.edu.tw; 5 Department of Medicinal and Applied Chemistry, Kaohsiung Medical University, Kaohsiung 80708, Taiwan; mychang@kmu.edu.tw; 6 Department of Chemistry, University of Illinois Urbana, Champaign, IL 61820, USA; chiehkai@illinois.edu; 7 School of Dentistry, College of Dental Medicine, Kaohsiung Medical University, Kaohsiung 80708, Taiwan; jhjeng@kmu.edu.tw; 8 Department of Dentistry, Kaohsiung Medical University Hospital, Kaohsiung 80708, Taiwan; 9 Department of Dentistry, National Taiwan University Hospital, Taipei 100225, Taiwan; 10 School of Post-Baccalaureate Medicine, Kaohsiung Medical University, Kaohsiung 80708, Taiwan; 11 Department of Radiation Oncology, Kaohsiung Medical University Hospital, Kaohsiung Medical University, Kaohsiung 80708, Taiwan; 12 Center for Cancer Research, Kaohsiung Medical University, Kaohsiung 80708, Taiwan; 13 Department of Medical Research, Kaohsiung Medical University Hospital, Kaohsiung 80708, Taiwan

**Keywords:** dioxabicyclo[3.3.1]nonane, oral cancer, radiosensitization, oxidative stress

## Abstract

This in vitro study examines the anti-oral cancer effects and mechanisms of a combined X-ray/SK2 treatment, i.e., X-ray and 6-*n*-butoxy-10-nitro-12,13-dioxa-11-azatricyclo[7.3.1.0^2,7^]trideca-2,4,6,10-tetraene (SK2). ATP cell viability and flow cytometry-based cell cycle, apoptosis, oxidative stress, and DNA damage assessments were conducted. The X-ray/SK2 treatment exhibited lower viability in oral cancer (Ca9-22 and CAL 27) cells than in normal (Smulow–Glickman, S-G) cells, i.e., 32.0%, 46.1% vs. 59.0%, which showed more antiproliferative changes than with X-ray or SK2 treatment. Oral cancer cells under X-ray/SK2 treatment showed slight subG1 and G2/M increments and induced high annexin V-monitored apoptosis compared to X-ray or SK2 treatment. The X-ray/SK2 treatment showed higher caspase 3 and 8 levels for oral cancer cells than other treatments. X-ray/SK2 showed a higher caspase 9 level in CAL 27 cells than other treatments, while Ca9-22 cells showed similar levels under X-ray and/or SK2. The X-ray/SK2 treatment showed higher reactive oxygen species (ROS) generation and mitochondrial membrane potential (MMP) depletion than other treatments. Meanwhile, the mitochondrial superoxide (MitoSOX) and glutathione levels in X-ray/SK2 treatment did not exhibit the highest rank compared to others. Moreover, oral cancer cells had higher γH2AX and/or 8-hydroxy-2-deoxyguanosine levels from X-ray/SK2 treatment than others. All these measurements for X-ray/SK2 in oral cancer cells were higher than in normal cells and attenuated by N-acetylcysteine. In conclusion, X-ray/SK2 treatment showed ROS-dependent enhanced antiproliferative, apoptotic, and DNA damage effects in oral cancer cells with a lower cytotoxic influence on normal cells.

## 1. Introduction

For oral cancers, radiation treatment can be either monotherapy or combined therapy in addition to surgery. Two common radiotherapies for oral cancer include external-beam radiation therapy (EBRT) [1] and chemoradiation [2]. EBRT, a high-energy X-ray irradiation treatment, is generally applied to patients with small tumors unsuitable for surgery [1]. Meanwhile, chemoradiation is a combined treatment with radiation and anticancer drugs such as cisplatin, but generally causes side effects [2].

Radioresistance occasionally occurs in oral cancer therapy, which handicaps its therapeutic effects [3,4]. Combining different treatments is a common strategy for solving radioresistant oral cancer [5]. Various naturally occurring and chemically synthesized compounds are employed to improve the sensitivity of cancerous tissues to radiotherapy [6,7,8,9], reducing the radioresistance. An optimal radiosensitizer is an anticancer agent that enhances radiotherapy’s effectiveness while incurring minimal damage to normal cells [10,11]. Continued research is urgently required to discover drugs that will provide reasonable radiosensitizers in oral cancer therapy.

A combined treatment, such as luteolin/γ-ray for lung cancer cells [12], β-apopicropodophyllin/γ-ray for colon cancer cells [13], and 1α,25(OH)_2_D_3_/X-ray for lung and ovarian cancer cells [14], shows improved radiosensitizing effects against several types of cancer cells. However, these studies lack any assessment of the combined treatment effects on normal cells. Consequently, the requirement for minimal damage to normal cells was not examined.

Many natural products and synthetic drugs contain dioxabicyclo[3.3.1]nonane skeleton [15,16,17,18,19]. Utilizing this benzo-fused dioxabicyclo[3.3.1]nonane skeleton, we previously synthesized a 6-*n*-butoxy-10-nitro-12,13-dioxa-11-azatricyclo[7.3.1.0^2,7^]trideca-2,4,6,10-tetraene [20], namely, SK2. SK2 exerts minimal damage on normal cells but highly suppresses the proliferation of oral cancer cells, i.e., has selective apoptosis and antiproliferative properties [21].

Various reactive oxygen species (ROS)-modulating drugs [22,23,24,25,26] were reported to enhance radiosensitization to cancer cells. This showed the potential of synergistic antiproliferation via a combined treatment (X-ray/ROS-modulating drug). However, although SK2 showed ROS-inducing ability on oral cancer cells, the influence of the combined treatment (X-ray/SK2) on the inhibition of proliferation was not addressed.

The goal of this study is to evaluate the antiproliferation effects of a combined treatment (X-ray/SK2) on oral cancer cells in parallel with the safety assessment of normal cell treatment. In addition to proliferation, the effects of oxidative stress and its continuous impact on cell cycle distribution, apoptosis, and DNA damage were explored to reveal the underlying antiproliferation effects of X-ray/SK2.

## 2. Materials and Methods

### 2.1. Cell Cultures and Chemicals

Two human oral cancer cell lines (Ca9-22 and CAL 27) were derived from the JCRB Cell Bank (Osaka, Japan) and ATCC (Manassas, VA, USA). Normal gingival epithelioid cells (Smulow–Glickman; S-G) were used for normal control cells [27,28,29]. Cells were cultured in DMEM/F12 medium (3:2; Gibco, Grand Island, NY, USA) and supplemented with 10% fetal bovine serum mixed with antibiotics at 37 °C with 5% CO_2_, as previously mentioned [30].

The SK2 was synthesized, as mentioned in our previous study [20], and dissolved in DMSO before use. *N*-acetylcysteine (NAC [31,32]; Sigma-Aldrich, St. Louis, MO, USA) pretreatment (10 mM, 1 h), a glutathione precursor, was applied to examine the oxidative stress in the combined treatment (X-ray/SK2).

### 2.2. X-ray Irradiation

With or without NAC pretreatment, the X-ray/SK2 combined treatment was performed. In brief, cells were received with X-ray (12 Gy) under the condition (1 Gy/min, 100 cm source–axis distance (SAD) by a 6 MV photon linear accelerator (Elekta Axesse, Stockholm, Sweden). Then, the cells were incubated in SK2 for 24 h. Non-irradiated cells received the mock treatments. The concentration of DMSO (0.1%) was the same in different treatments (control, SK2, X-ray, or X-ray/SK2).

### 2.3. Cell Viability

An ATP measurement kit (PerkinElmer Life Sciences, Boston, MA, USA) was used to evaluate cell viability, as previously mentioned [33,34,35,36].

### 2.4. Cell Cycle

Cells were subjected to a 75% ethanol fixation overnight and then washed and incubated with 7-aminoactinomycin D (7AAD; Biotium Inc., Hayward, CA, USA), i.e., 1 μg/mL 7AAD, for 30 min [37]. The cells were re-mixed in PBS and conducted to the analysis using Guava easyCyte flow cytometry (Luminex, Austin, TX, USA).

### 2.5. Apoptosis (Annexin V/7AAD)

Cells were mixed with annexin V-FITC (Strong Biotech Corp., Taipei, Taiwan)/7AAD, in quantities of 10 and 1 μg/mL, respectively, for 30 min [30,38]. Then, cells were re-mixed in PBS and conducted to flow cytometry.

### 2.6. Apoptosis Signaling (Caspases 3/8/9)

Caspase 3/8/9 activities were analyzed using flow cytometry [39]. PhiPhiLux-G1D2, CaspaLux8-L1D2, and CaspaLux9-M1D2 (OncoImmunin, Gaithersburg, MD, USA) are caspase 3-, 8-, and 9-targeting peptides that are cleaved by activated caspases 3, 8, and 9 when cells become apoptotic. After cleavage, the cleaved (activated) caspase 3-, 8-, and 9-targeting peptides become fluorophores. This fluorescence intensity is proportional to the degree of caspase 3, 8, and 9 activation. In brief, 10 μM caspase 3/8/9 substrates were incubated with cells at 37 °C for 1 h. Then, cells were re-mixed in PBS and conducted to flow cytometry.

### 2.7. Cellular ROS, Mitochondrial Superoxide (MitoSOX), Mitochondrial Membrane Potential (MMP), and Cellular Glutathione (GSH)

ROS and MitoSOX are sensitive to reaction with 2′,7′-dichlorodihydrofluorescein diacetate (DCFH-DA; Molecular Probes, Invitrogen, Eugene, OR, USA; 10 μM, 30 min) [40] and MitoSOX™ Red [41] (Sigma-Aldrich; 50 nM, 30 min). MMP and GSH were detected through staining with DiOC_2_(3) [41] (Invitrogen, San Diego, CA, USA; 5 nM, 30 min) and 5-chloromethylfluorescein diacetate (CMF-DA [30]; 5 μM, 20 min; Thermo Fisher Scientific, Carlsbad, CA, USA). After the reactions, these stained cells were washed and re-mixed in PBS for flow cytometry, as previously mentioned [30,41].

### 2.8. DNA Damage (γH2AX and 8-Hydroxy-2-Deoxyguanosine (8-OHdG))

γH2AX and 8-OHdG are typical biomarkers for DNA double-strand breaks and oxidative DNA damage and are detectable using flow cytometry, as mentioned previously [30]. In brief, they were detected using antibodies such as p-Histone H2A.X (Ser 139) mAb and 8-OHdG antibody (Santa Cruz Biotechnology, Santa Cruz, CA, USA).

### 2.9. Statistical Analysis

Significant differences for multi-comparison were examined with JMP 12 software (SAS Institute, Cary, NC, USA), choosing one-way analysis of variance (ANOVA) and the Tukey HSD Test. Different groups were assigned with small notes by JMP 12. It shows significant results when different groups exhibit non-overlapping notes (*p* < 0.05).

## 3. Results

### 3.1. Cell Viability of X-ray and/or SK2 Treatments in Oral Cancer and Normal Cells

**Figure 1:**
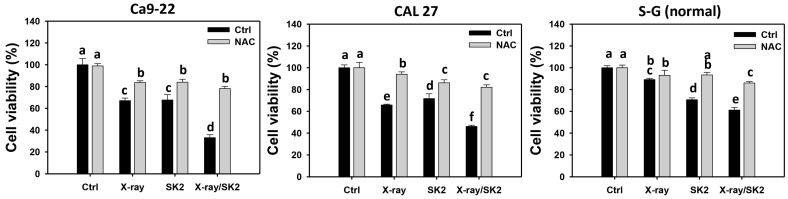
Cell viability of X-ray and/or SK2 treatments. Cells (cancer and normal) were treated with X-ray and/or SK2 (12 Gy; 10 µg/mL) for 24 h. NAC pretreatment was applied to confirm oxidative stress. Data, mean ± SD (*n* = 3). Significant results occur when different groups exhibit non-overlapping notes (*p* < 0.05). These notes were assigned using JMP statistic software for multi-comparisons, as mentioned in Section 2.9. For the example of Ca9-22 cells, X-ray/SK2 significantly differs from others because the notes are different, i.e., d vs. a, c, and c for X-ray/SK2, control, X-ray, and SK2. Meanwhile, the X-ray and SK2 show no significant difference because the note is the same (overlapped).

As shown in Figure 1, combined treatments of X-ray (12 Gy) and SK2 (10 μg/mL), namely, X-ray/SK2, displayed lower cell viability of oral cancer cells than single treatment with X-ray or SK2, i.e., 32.0% vs. 67.1% and 67.7% (Ca9-22) and 46.1% vs. 65.8% and 71.6% (CAL 27), respectively. Moreover, X-ray/SK2 exhibited lower oral cancer cell viability than normal cells, i.e., 32.0%, 46.1% vs. 59.0% (Ca9-22, CAL 27 vs. S-G), respectively. Accordingly, X-ray/SK2 exhibited a high antiproliferative function in oral cancer cells, with a low influence on normal cells.

Moreover, all the cell viability of the X-ray and/or SK2 treatments was partly recovered with NAC pretreatment (Figure 1). This change suggests that oxidative stress mediated the enhanced antiproliferative ability of X-ray/SK2.

### 3.2. Cell Cycle Perturbance of X-ray and/or SK2 Treatments in Oral Cancer and Normal Cells

**Figure 2:**
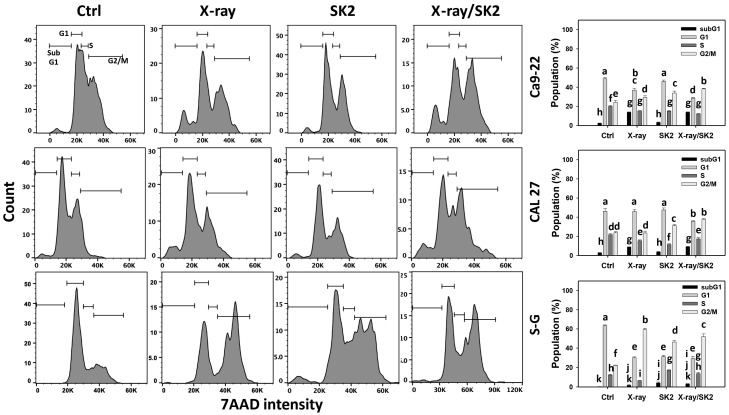
Cell cycle distribution of X-ray and/or SK2 treatments. Cells (cancer and normal) were treated with X-ray and/or SK2 (12 Gy; 10 µg/mL) for 24 h. NAC pretreatment was applied to confirm oxidative stress. Data, mean ± SD (*n* = 3). Significant results occur when different groups exhibit non-overlapping notes (*p* < 0.05). These notes were assigned using JMP statistic software for multi-comparisons, as mentioned in Section 2.9.

As shown in Figure 2, X-ray/SK2 and X-ray treatments of oral cancer cells exhibited higher subG1 than others (control and SK2). For the oral cancer cells, X-ray/SK2 exhibited lower G1 and higher G2/M phases of oral cancer cells than the others (control, X-ray, and SK2). In comparison, normal cells (S-G) showed weak subG1 for all treatments and lower G1 and higher G2/M phases in X-ray and/or SK2 than the control. Accordingly, X-ray/SK2 exhibited high subG1 accumulation in oral cancer cells, with a low influence on normal cells.

### 3.3. Annexin V-Apoptosis and Caspase 3 Activation of X-ray and/or SK2 Treatments in Oral Cancer and Normal Cells

**Figure 3:**
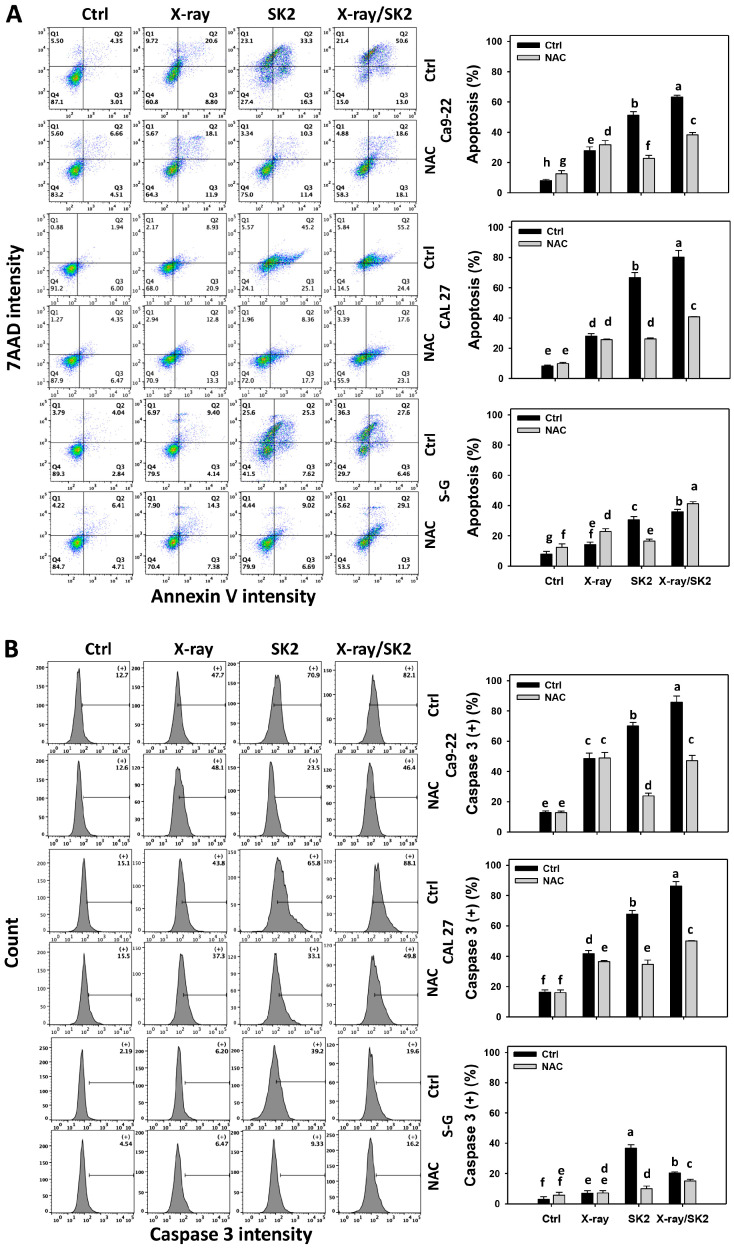
Annexin V and caspase 3 levels of X-ray and/or SK2 treatments. Cells (cancer and normal) were treated with X-ray and/or SK2 (12 Gy; 10 µg/mL) for 24 h. NAC pretreatment was applied to confirm oxidative stress. (**A**) Pattern and statistics of annexin V/7AAD analyses for X-ray and/or SK2 treatments. Annexin V (+)/7AAD (+ or −) is counted as apoptosis (+) (%) [42]. (**B**) Pattern and statistics of caspase 3 analyses for X-ray and/or SK2 treatments. (+) is counted as high intensity (+) (%). Data, mean ± SD (*n* = 3). Significant results occur when different groups exhibit non-overlapping notes (*p* < 0.05). These notes were assigned using JMP statistic software for multi-comparisons, as mentioned in Section 2.9.

Annexin V/7AAD and caspase 3 activation were conducted using flow cytometry to assess the apoptosis [30]. For cancer cells and normal (S-G) cells, X-ray/SK2 treatment exhibited higher apoptosis (+) (%) than the others (Figure 3A). For oral cancer cells, X-ray and/or SK2 treatments exhibited higher activated caspase 3 (+) (%) than the others (Figure 3B). X-ray/SK2 and SK2 treatments for S-G cells exhibited higher caspase 3 (+) (%) than the others.

Notably, oral cancer cells exhibited higher apoptosis (annexin V) (+) (%) from X-ray/SK2 treatment than normal cells, i.e., 63.6%, 79.6% vs. 34.1% for Ca9-22, CAL 27 vs. S-G, respectively (Figure 3A). Oral cancer cells exhibited higher activated caspase 3 (+) (%) from X-ray/SK2 treatment than normal cells, i.e., 82.1%, 88.1% vs. 19.6% for Ca9-22, CAL 27 vs. S-G (Figure 3B). Accordingly, X-ray/SK2 exhibited higher apoptosis and caspase 3 activation in oral cancer cells, with a low influence on normal cells.

Moreover, all the apoptosis and caspase 3 activation of the X-ray and/or SK2 treatments were attenuated with the NAC pretreatment (Figure 3A,B). This change suggests that oxidative stress mediated the enhanced apoptosis and caspase 3 activation of X-ray/SK2.

### 3.4. Extrinsic and Intrinsic Caspases of X-ray and/or SK2 Treatments in Oral Cancer and Normal Cells

**Figure 4:**
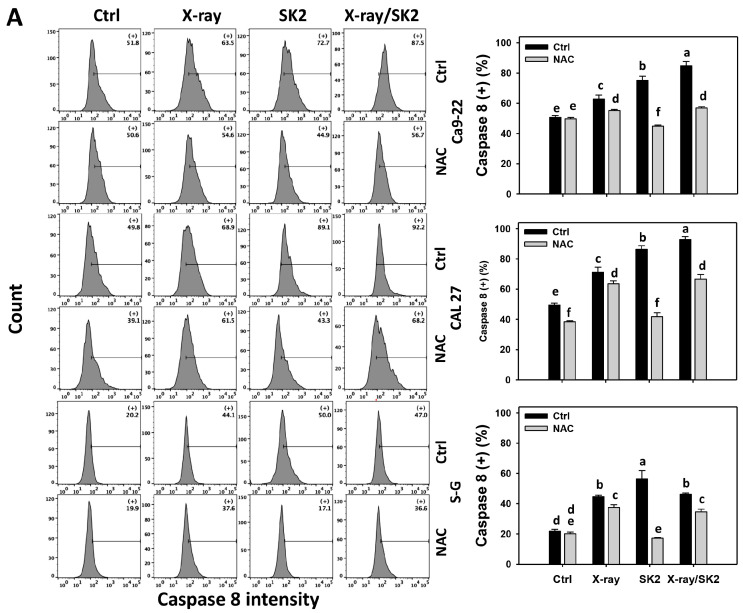

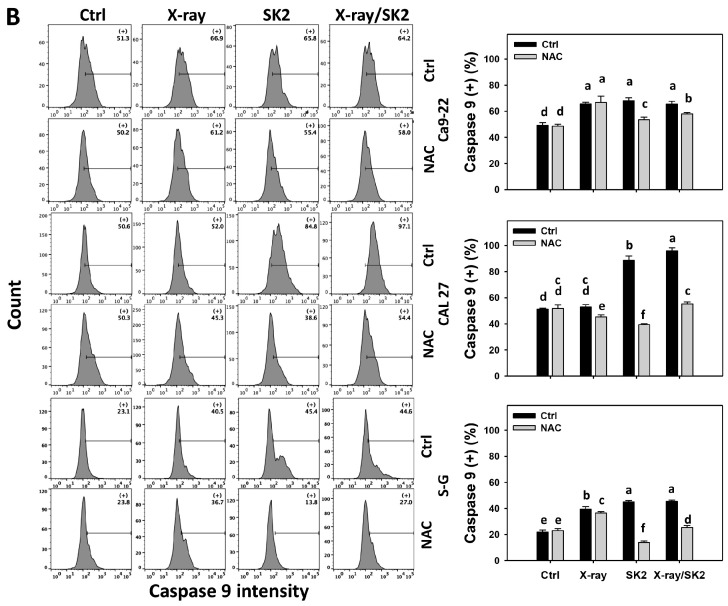
Activated caspase 8 and 9 levels of X-ray and/or SK2 treatments. Cells (cancer and normal) were treated with X-ray and/or SK2 (12 Gy; 10 µg/mL) for 24 h. NAC pretreatment was applied to confirm oxidative stress. (**A**,**B**) Patterns and statistics of activated caspase 8 and 9 analyses for X-ray and/or SK2 treatments. (+) is counted as high intensity (+) (%) of activated caspases 8 and 9. Data, mean ± SD (*n* = 3). Significant results occur when different groups exhibit non-overlapping notes (*p* < 0.05). These notes were assigned using JMP statistic software for multi-comparisons, as mentioned in Section 2.9.

To clarify the participation of extrinsic and intrinsic apoptosis, the activations of caspases 8 and 9 were evaluated [43]. Oral cancer cells exhibited higher activated caspase 8 (+) (%) with X-ray/SK2 treatment than with the others (Figure 4A). For normal (S-G) cells, X-ray and/or SK2 treatments exhibited higher activated caspase 8 (+) (%) than the control (Figure 4A). Notably, X-ray/SK2 treatment exhibited higher activated caspase 8 (+) (%) in oral cancer cells than in normal cells, i.e., 87.5%, 92.2% vs. 47.0% for Ca9-22, CAL 27 vs. S-G, respectively.

Similarly, for cancer (Ca9-22) and normal (S-G) cells, the X-ray and/or SK2 treatment exhibited higher activated caspase 9 (+) (%) than the control (Figure 4B). For CAL 27 cells, X-ray/SK2 and SK2 treatments exhibited higher activated caspase 9 (+) (%) than the others (Figure 4B). Notably, X-ray/SK2 treatment exhibited higher activated caspase 9 (+) (%) in oral cancer cells than in normal cells, i.e., 60.0%, 95.7% vs. 45.0% for Ca9-22, CAL 27 vs. S-G, respectively.

Moreover, all the caspase 8 and 9 activations of X-ray and/or SK2 treatments were attenuated with the NAC pretreatment (Figure 4A,B). This change suggests that oxidative stress mediated the enhanced apoptosis and the caspase 8 and 9 activations of X-ray/SK2.

### 3.5. ROS and MitoSOX Levels of X-ray and/or SK2 Treatments in Oral Cancer and Normal Cells

**Figure 5:**
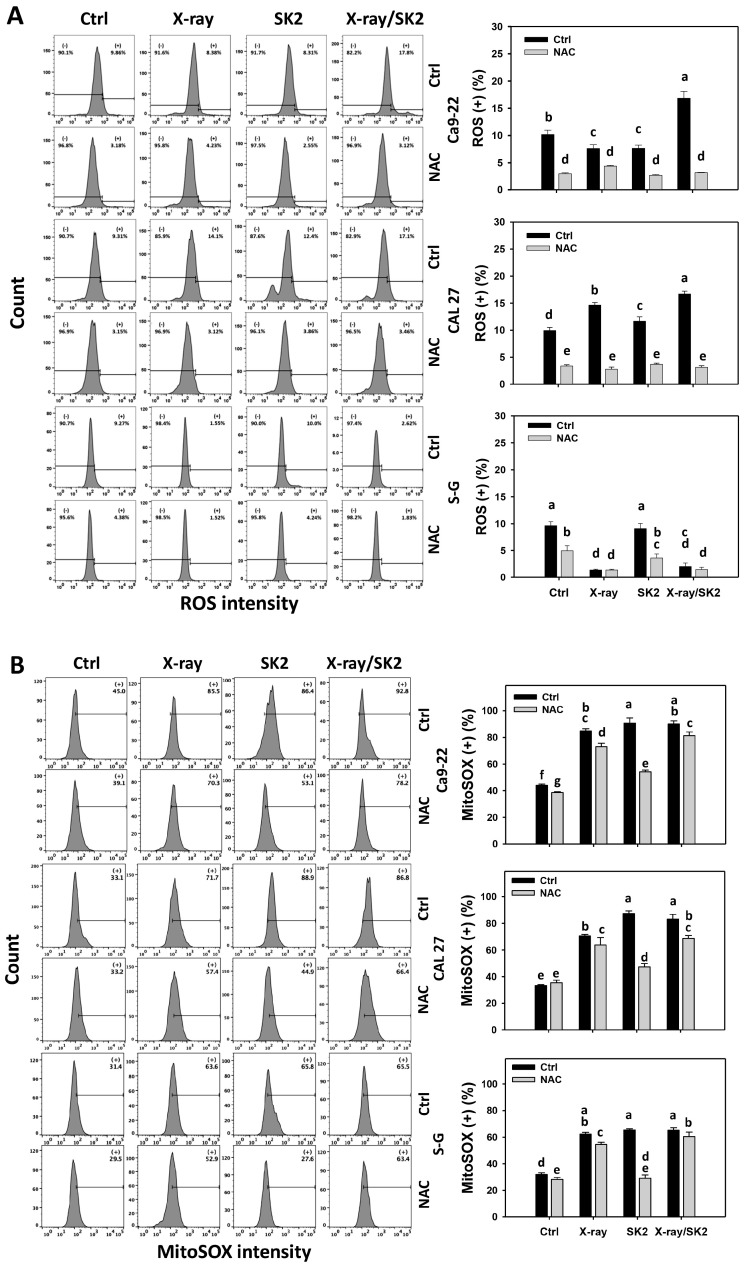
ROS and MitoSOX levels of X-ray and/or SK2 treatments. Cells (cancer and normal) were treated with X-ray and/or SK2 (12 Gy; 10 µg/mL) for 24 h. NAC pretreatment was applied to confirm oxidative stress. (**A**,**B**) Pattern and statistics of ROS and MitoSOX analyses for X-ray and/or SK2 treatments. (+) is counted as high intensity (+) (%). Data, mean ± SD (*n* = 3). Significant results occur when different groups exhibit non-overlapping notes (*p* < 0.05). These notes were assigned using JMP statistic software for multi-comparisons, as mentioned in Section 2.9.

To clarify the participation of cellular and mitochondrial oxidative stress, ROS and MitoSOX were evaluated [43]. Cancer cells exhibited higher ROS (+) (%) with the X-ray/SK2 treatment than with the others (Figure 5A). In comparison, normal (S-G) cells displayed a lower ROS level with the X-ray/SK2 treatment than with the others. Notably, oral cancer cells exhibited higher ROS (+) (%) from the X-ray/SK2 treatment than normal cells, i.e., 16.5%, 17.1% vs. 2.3% for Ca9-22, CAL 27 vs. S-G, respectively.

Furthermore, the X-ray/SK2 treatment for cancer and normal cells exhibited higher MitoSOX (+) (%) than the control (Figure 5B). Notably, oral cancer cells exhibited higher MitoSOX (+) (%) from the X-ray/SK2 treatment than normal cells, i.e., 89.1%, 82.3% vs. 65.0% for Ca9-22, CAL 27 vs. S-G, respectively.

All the ROS and MitoSOX levels of X-ray and/or SK2 treatments were attenuated with the NAC pretreatment (Figure 5A,B). This change suggests the enhanced ROS and MitoSOX of X-ray/SK2 were mediated via oxidative stress.

### 3.6. MMP and GSH Levels of X-ray and/or SK2 Treatments in Oral Cancer and Normal Cells

**Figure 6:**
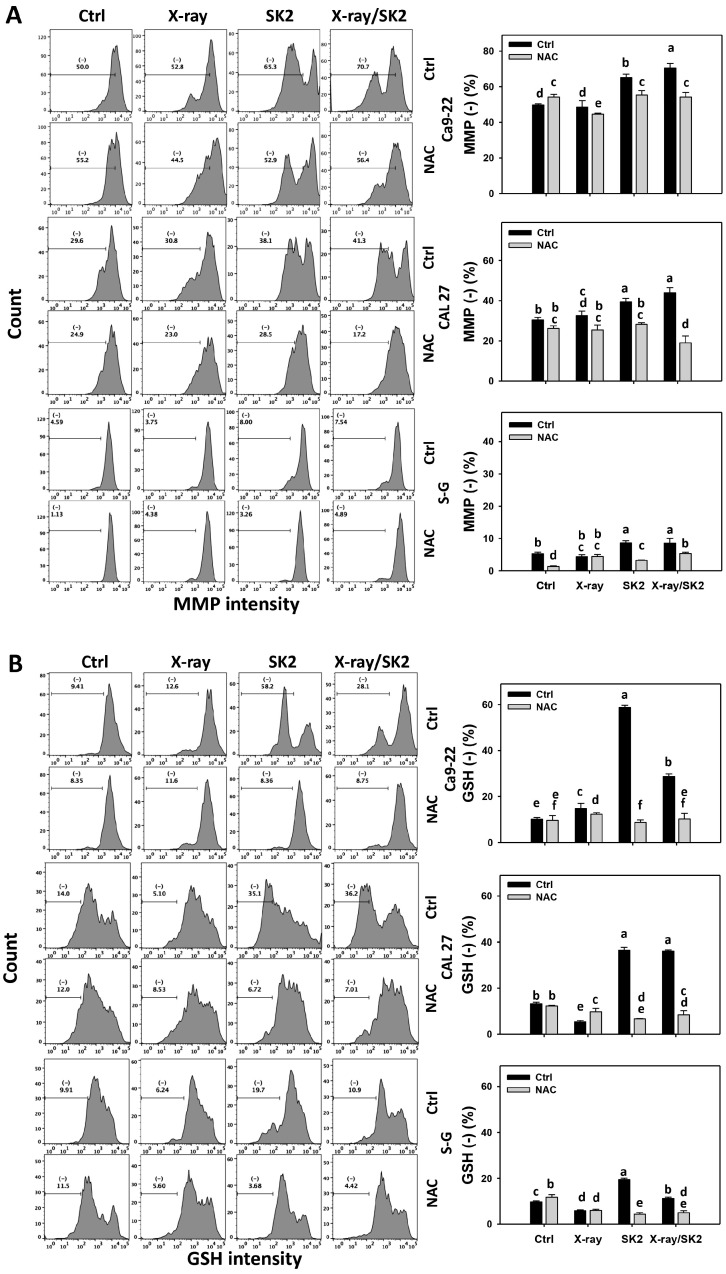
MMP and GSH levels of X-ray and/or SK2 treatments. Cells (cancer and normal) were treated with X-ray and/or SK2 (12 Gy; 10 µg/mL) for 24 h. NAC pretreatment was applied to confirm oxidative stress. (**A**,**B**) Pattern and statistics of MMP and GSH analyses for X-ray and/or SK2 treatments. (−) is counted as low intensity (−) (%). Data, mean ± SD (*n* = 3). Significant results occur when different groups exhibit non-overlapping notes (*p* < 0.05). These notes were assigned using JMP statistic software for multi-comparisons, as mentioned in Section 2.9.

MMP and GSH depletion enhances oxidative stress [43]; therefore, their involvement in regulating oxidative stress was assessed. For cancer cells, the X-ray/SK2 treatment exhibited higher MMP (−) (%) than the others (Figure 6A). In comparison, normal (S-G) cells showed a weakly higher MMP (−) with X-ray/SK2 and SK2 treatment than with the others. Notably, oral cancer cells exhibited higher MMP (−) (%) from the X-ray/SK2 treatment than normal cells, i.e., 70.1%, 41.3% vs. 8.5% for Ca9-22, CAL 27 vs. S-G, respectively.

Furthermore, the X-ray/SK2 and SK2 treatments for cancer cells exhibited higher GSH (−) (%) than the control (Figure 6B). Notably, the X-ray/SK2 treatment exhibited higher GSH (−) (%) in oral cancer cells than in normal cells, i.e., 27.9%, 36.5% vs. 10.5% for Ca9-22, CAL 27 vs. S-G, respectively.

All the MMP and GSH (−) levels of X-ray and/or SK2 treatments were attenuated with the NAC pretreatment (Figure 6A,B). This change suggests that oxidative stress promoted the enhanced ROS and MitoSOX depletion of X-ray/SK2 treatment.

### 3.7. DNA Damage Levels of X-ray and/or SK2 Treatments in Oral Cancer and Normal Cells

**Figure 7:**
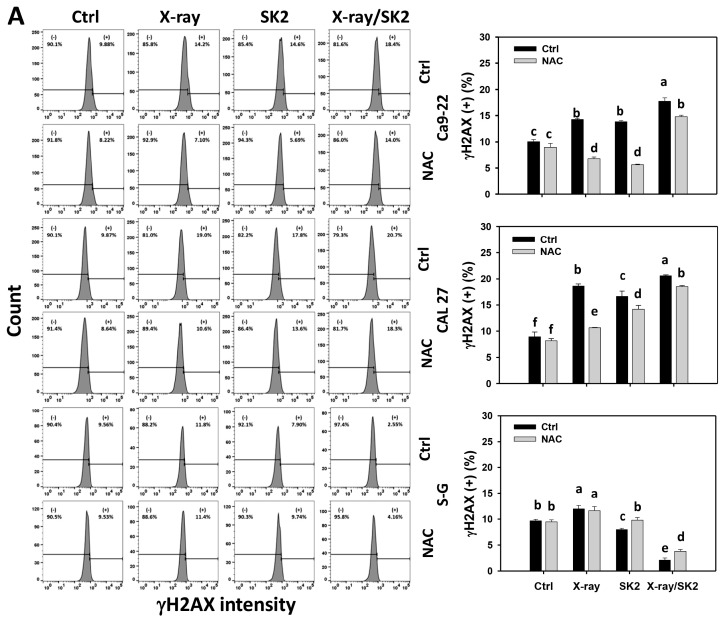

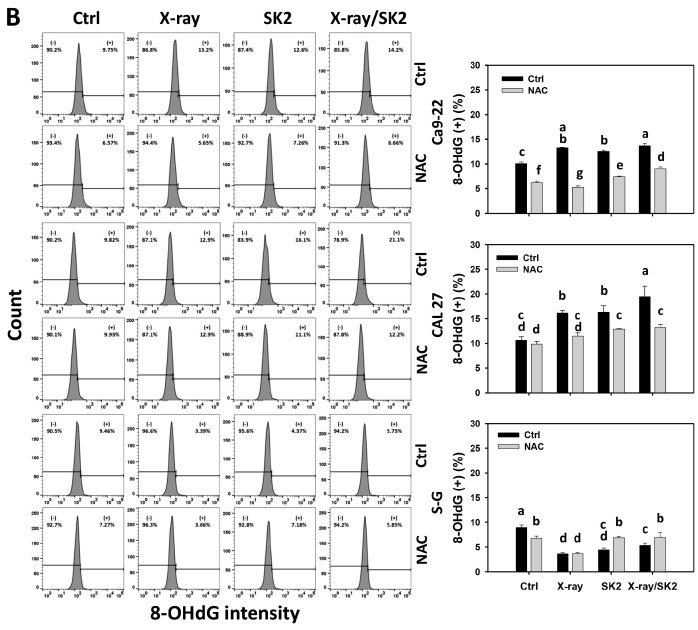
DNA damage levels of X-ray and/or SK2 treatments. Cells (cancer and normal) were treated with X-ray and/or SK2 (12 Gy; 10 µg/mL) for 24 h. NAC pretreatment was applied to confirm oxidative stress. (**A**,**B**) Pattern and statistics of γH2AX and 8-OHdG analyses for X-ray and/or SK2 treatments. The dashed-line region is high intensity (+) (%). Data, mean ± SD (*n* = 3). Significant results occur when different groups exhibit non-overlapping notes (*p* < 0.05). These notes were assigned by JMP statistic software for multi-comparisons, as mentioned in Section 2.9.

To clarify the participation of DNA damage, γH2AX and 8-OHdG were evaluated [43]. Oral cancer cells exhibited higher γH2AX (+) (%) from X-ray/SK2 treatment than others (Figure 7A). In comparison, normal (S-G) cells displayed a lower γH2AX level with X-ray/SK2 treatment than with the others. Notably, the X-ray/SK2 treatment exhibited higher γH2AX (+) (%) in oral cancer cells than normal cells, i.e., 17.5%, 20.3% vs. 1.6% for Ca9-22, CAL 27 vs. S-G, respectively.

Furthermore, X-ray and/or SK2 treatments for cancer cells exhibited higher 8-OHdG (+) (%) than the control (Figure 7B). Notably, oral cancer cells exhibited higher 8-OHdG (+) (%) from X-ray/SK2 treatment than normal cells, i.e., 13.4%, 19.7% vs. 5.0% for Ca9-22, CAL 27 vs. S-G, respectively.

All the γH2AX and 8-OHdG levels of X-ray and/or SK2 treatments in oral cancer cells were attenuated with the NAC pretreatment (Figure 7A,B). This change suggests the enhanced γH2AX and 8-OHdG of X-ray/SK2 treatment were mediated via oxidative stress.

## 4. Discussion

X-ray/SK2 treatment exhibited an enhanced antiproliferative function on oral cancer cells rather than on normal cells. The difference of X-ray/SK2 between Ca9-22/CAL 27 and normal cell (S-G) viability was 27.0% (Ca9-22 vs. S-G) and 12.9% (CAL 27 vs. S-G). The potential mechanism of enhanced antiproliferation in X-ray/SK2 treatment is discussed as follows.

### 4.1. Role of Oxidative Stress in Enhanced Antiproliferative Effects of X-ray/SK2

X-ray induces oxidative stress [44], causing apoptosis [45]. ROS-modulating anticancer drugs [46,47] trigger oxidative stress. Therefore, combined ROS-modulating drugs with X-rays have the potential for enhanced antiproliferative effects. For example, luteolin/γ-ray treatment improves radiosensitivity through the induction of high ROS and apoptosis in lung cancer cells [12]. β-apopicropodophyllin also enhances γ-ray radiosensitivity by triggering ROS and apoptosis in colon cancer cells [13]. The X-ray radiosensitivity of lung and ovarian cancer cells is enhanced by 1α,25(OH)_2_D_3_ through the induction of ROS-mediated apoptosis [14].

Similarly, oral cancer cells possess higher oxidative stress levels than normal cells [21]. Hence, higher oxidative stress levels are anticipated from X-ray/SK2 than from single treatments. In the present study, oxidative stress factors such as ROS, MitoSOX, and GSH levels were considered. Oral cancer cells had higher ROS production and MMP depletion from X-ray/SK2 than from a single treatment (Figure 5). In comparison, the X-ray/SK2 and SK2 showed similar levels of MitoSOX, although they were higher than the other treatments in oral cancer cells.

Compared to normal cells, this high oxidative stress ability of X-ray/SK2 was consistent with the high apoptosis in oral cancer cells. Moreover, oral cancer cells had higher caspase 3 and 8 levels following X-ray/SK2 compared to with the other treatments. CAL 27 cells showed higher caspase 9 levels with X-ray/SK2 treatment compared to with the other treatments, whereas Ca9-22 cells show similar caspase 9 levels in X-ray and/or SK2 treatments. This suggests different oral cancer cells have a differential induction for some caspase signaling in X-ray/SK2 treatment.

### 4.2. The Impact of DNA Damage and Repair by Radiosensitizers

X-ray stimulates DNA damage, such as γH2AX [48] and 8-OHdG [49]. This DNA induction was consistent in the present study using oral cancer cells. In comparison, normal cells showed lower DNA damage than oral cancer cells in X-ray treatment. Furthermore, SK2 also induced greater γH2AX and 8-OHdG expression in oral cancer cells than in normal cells [21]. X-ray/SK2 showed higher γH2AX levels in Ca9-22 cells, and additionally, it showed higher γH2AX and 8-OHdG levels in Ca9-22 cells compared to other treatments (Figure 7). All of this X-ray/SK2-caused DNA damage was higher in oral cancer cells than in normal cells. Therefore, X-ray/SK2 shows enhanced DNA damage in oral cancer cells, although they have a differential induction for different DNA damage types.

Some radiosensitizers, such as enterolactone [6], quercetin [8], and garcinol [50], exhibit DNA repair-suppressing effects to improve the radiosensitivity of breast, ovarian, and cervical cancer cells. Some anticancer drugs, such as physapruin A, exhibit DNA damage-promoting and repair-suppressing impacts on oral cancer cells [51]. Normal cells may show lower DNA damage due to their proficient DNA repair ability. It warrants an assessment of the DNA repair impact on X-ray/SK2-treated oral cancer cells.

### 4.3. Potential Role of G2/M arrest in X-ray/SK2 Treatment

G2/M arrest is one of the sensitivity markers of radiotherapy [52]. X-ray radiation causes a G2/M arrest in various cancer cells, such as lung (A549) [53], oral (SAS), melanoma (C32TG), and lymphoma cells [54]. Cancer cells in the G2/M phase are the most radiosensitive compared to those in other phases [55,56]. Irinotecan alone induces G2/M arrest, and the combined treatment (X-ray/irinotecan) induces more G2/M arrest and apoptosis than irinotecan alone in colon cancer (HT29) cells [57]. Similarly, SK2 caused G2/M arrest, which was further enhanced in X-ray/SK2 of oral cancer cells (Figure 2).

### 4.4. Limitation of the Present Study and Future Prospects

Except for apoptosis, the present study did not evaluate non-apoptotic impacts, such as endoplasmic reticulum stress, necroptosis, autophagy, or ferroptosis [58] modulated by ROS [59]. Non-apoptotic death can overcome the potential resistance and impairment to cancer cell apoptosis [60]. Consequently, the involvement of non-apoptotic death effects and the mechanism of the oxidative stress-dependent enhanced antiproliferation of X-ray/SK2 treatment need to be thoughtfully investigated in the future.

The present study only relied on the in vitro evidence of the combined effects of X-ray/SK2 on oral cancer, with little toxicity of normal cells. In vivo evidence is now required to enhance the promise of this combined treatment before testing its clinical application. Concerning primary drug safety, animal administration of X-ray/SK2 is essential to confirm no toxic effects in addition to the normal cell model evidence. Next, clinical trials of X-ray/SK2 in healthy volunteers or oral cancer patients are required for further systematic clinical evaluation of the treatment.

## 5. Conclusions

This study validated the radiosensitization and treatment safety of benzo-fused dioxabicyclo[3.3.1]nonane derivative (SK2) by testing two oral cancer cell lines and one normal cell line. X-ray/SK2 displayed enhanced antiproliferation of oral cancer cells, accompanied by increasing ROS, apoptosis, caspase signaling, and DNA damage, which were higher than in normal cells. These X-ray/SK2-induced changes were attenuated with NAC, revealing that oxidative stress-dependent regulation was involved. Therefore, X-ray/SK2 is an effective anti-oral cancer treatment considering the safety concern to normal cells inherent in other treatments.

## Data Availability

Data is contained within the article.
